# Peripherally Embolizing Aortic Thrombus: The Work-Up, Management, and Outcome of Primary Aortic Thrombus

**DOI:** 10.1155/2019/8132578

**Published:** 2019-07-02

**Authors:** Ramy Mando, Robert Gemayel, Ashish Chaddha, Julian J. Barbat, Elvis Cami

**Affiliations:** ^1^Department of Internal Medicine, Beaumont Health System, Royal Oak, MI, USA; ^2^Michigan State University College of Osteopathic Medicine, Lansing, MI, USA; ^3^Department of Cardiovascular Medicine, Beaumont Health System, Royal Oak, MI, USA

## Abstract

**Background:**

Primary aortic thrombus is an uncommon entity and not frequently reported in the literature. Herein, we discuss the presentation and management of a patient with a primary thoracic mural thrombus.

**Case Summary:**

A 46-year-old female with past medical history of tobacco dependence presented for low-grade fever and sudden onset, severe right upper quadrant abdominal pain with associated nausea and vomiting. Computed tomography (CT) revealed an intraluminal polypoid filling defect arising from the isthmus of the aorta projecting into the proximal descending aorta and findings consistent with infarction of the spleen and right kidney. Infectious, autoimmune, hematologic, and oncologic work-up were all unyielding. The patient was started on heparin and later transitioned to apixaban 5 mg twice a day and 81 mg of aspirin daily. She was also counseled regarding smoking cessation. Two months follow-up CT revealed resolution of the thrombus. Patient had no further thromboembolic complications.

**Discussion:**

We present a unique case of primary aortic thrombus. To our knowledge, this is the first reported case managed successfully with a NOAC. This diagnosis is one of exclusion and through work-up should be completed. Our aim is to raise awareness of this condition and successful management with apixaban in low-risk patients.

## 1. Background

Aortic mural thrombi are uncommon and typically associated with severe atherosclerosis or the presence of an aneurysm. Even more uncommon is the formation of a primary aortic thrombus with no infectious, neoplastic, traumatic, autoimmune, or hypercoagulable triggers responsible for thrombus formation. To date, there is no consensus on the management of this condition; however, options include anticoagulation, thrombolytics, aortic surgery, and thromboaspiration. Herein, we describe a rare case of primary aortic thrombus presenting with systemic embolization to the lower extremities, kidney, and spleen. This is one of the first cases in the literature describing effective management of this condition with a direct oral anticoagulant.

## 2. Introduction

Thrombi in the thoracic aorta are most commonly associated with severe atherosclerosis or aortic aneurysm [1, 2]. Primary thrombus of the aorta in the absence of aneurysm, moderate-to-severe atherosclerosis, hypercoagulability, malignancy, infections, or autoimmune conditions is exceedingly rare [2–4]. Management of primary aortic thrombus is not well defined at this time [5, 6]. Herein, we describe the rare case of a thoracic aortic thrombus with peripheral embolization presenting as abdominal pain and lower extremity pain. This is one of the first cases in the literature describing the use of direct oral anticoagulants in the management of primary aortic thrombi. We aim to raise awareness of this condition and summarize pathophysiology as well as current treatment options available to us.

## 3. Case Description

### 3.1. Presentation and Initial Evaluation

**Figure 1:**
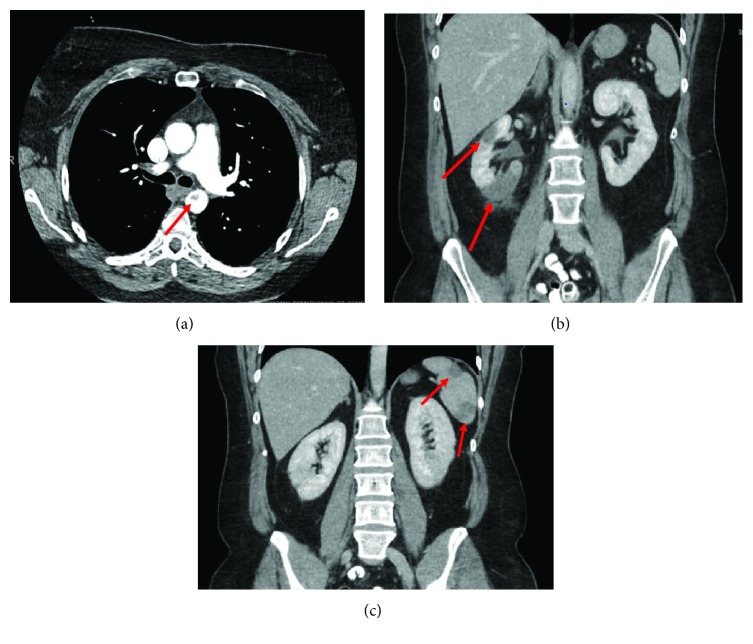
CT of the abdomen and pelvis revealing a mass in the descending thoracic aortia (a) with wedge-shaped infarctions in the right kidney (b) and spleen (c) consistent with an embolic phenomona.

A 46-year-old female with past medical history of tobacco dependence presented for low-grade fever and sudden onset of severe right upper quadrant abdominal pain with associated nausea and vomiting. Computed tomography (CT) completed in the emergency department revealed an intraluminal polypoid filling defect arising from the isthmus of the aorta projecting into the proximal descending aorta and findings consistent with infarctions of the spleen and right kidney (Figures 1(a)–1(c)). Given the fever, there was a concern for sepsis so she was started on broad-spectrum antibiotics. Heparin has also initiated cardioembolic phenomena of unclear etiology; however, it was later discontinued to prevent hemorrhagic transformation of the existing infarcts. The patient was admitted for further evaluation.

### 3.2. Inpatient Evaluation

**Figure 2:**
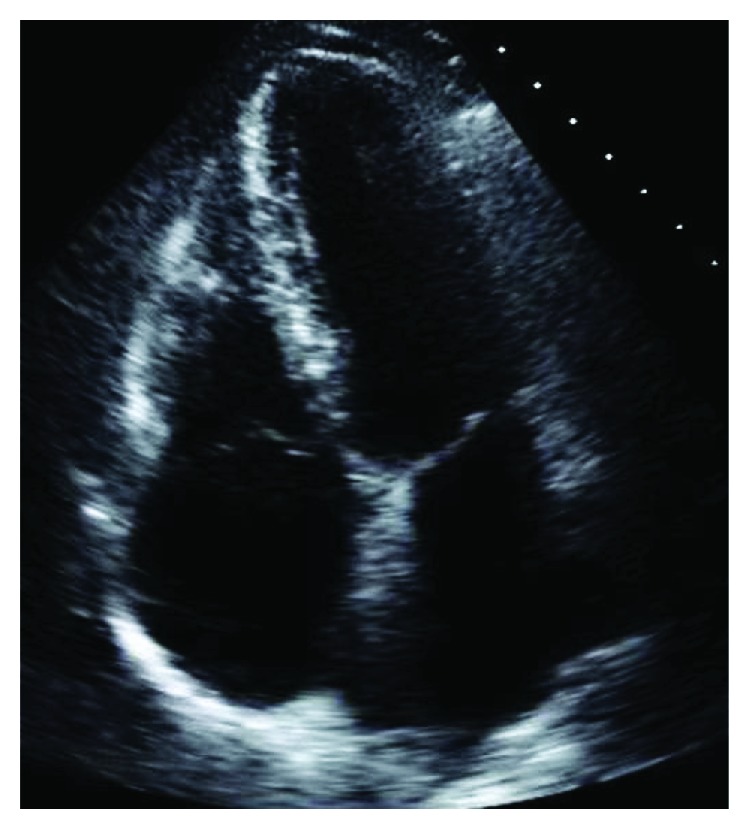
Apical four-chamber view of the heart with no evidence of a source of thrombosis.

**Figure 3:**
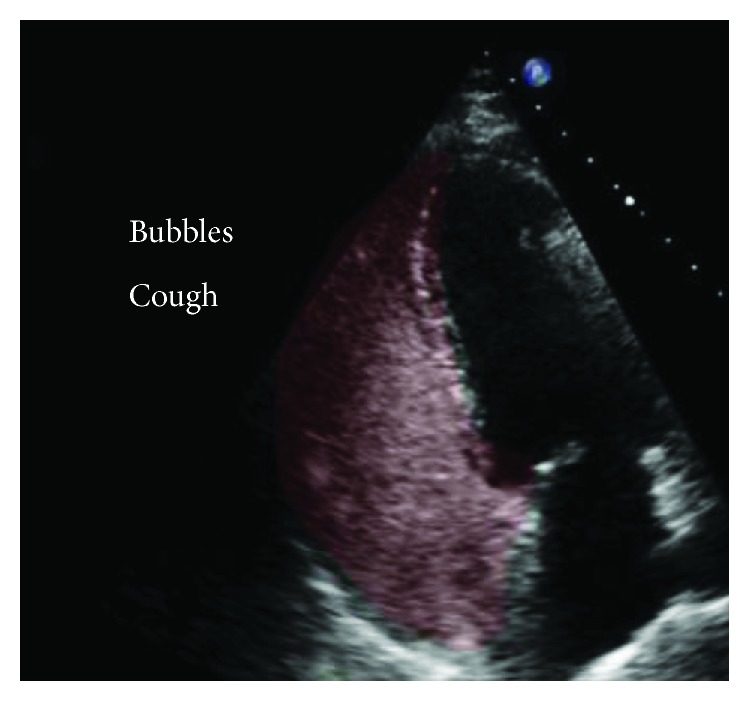
Agitated saline (highlighted in red) in the right heart revealing no crossover into the left heart consistent with no intracardiac shunts.

**Figure 4:**
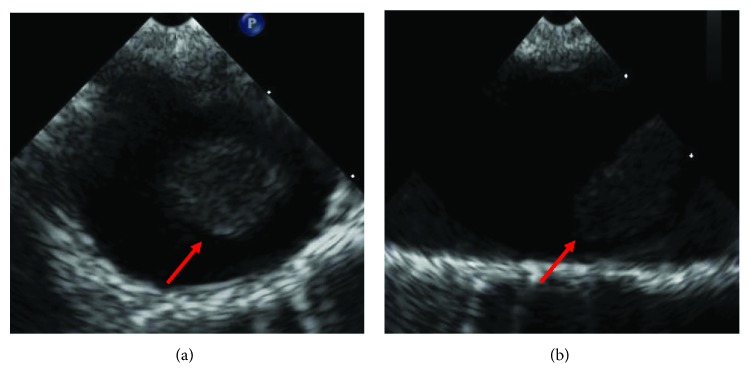
(a) Transesophageal echocardiogram (transverse view) of a large mass attached to the wall of the descending thoracic aorta. (b) Transesophageal echocardiogram (longitudinal view) of the large mobile mass attached to the aortic wall.

**Figure 5:**
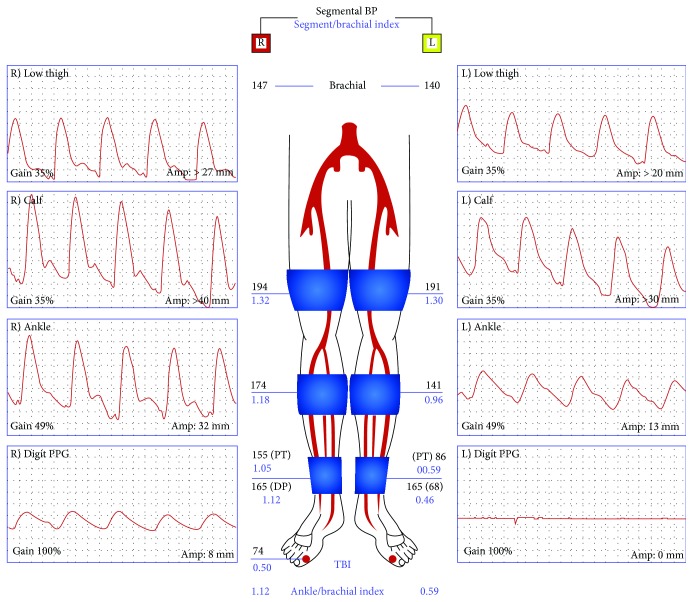
Lower extremity arterial Doppler with significant flow limitations in the left lower extremity consistent with diffuse obstructive atherosclerotic obstructive disease in the left posterior tibial artery, peroneal artery, and anterior tibial artery. The waveforms of the right lower extremity are normal multiphasic Doppler waveforms. In the left extremity, we see dampened monophasic Doppler flow signals in the area of the left posterior tibial artery and dorsalis pedis artery. Diminished pulse volume recordings were seen in the left ankle. Flat line, nonpulsatile flow signals were seen in the digits of the left foot.

A transthoracic echocardiogram showed no findings to suggest valvular or cardiac source of embolus (Figure 2). An agitated saline bubble contrast study was performed with no evidence of intracardiac shunting (Figure 3). A transesophageal echocardiogram was then completed and revealed a large echogenic mobile mass attached to the wall of the descending thoracic aorta (Figures 4(a) and 4(b)). Bilateral lower extremity arterial Dopplers were obtained and revealed severe popliteal-tibial arterial disease in the left lower extremity (Figure 5).

Work-up for other organic causes was extensive. Infectious work-up was negative and included testing for bacterial infection, fungal infection, HIV, Bartonella henselae antibody, Coxiella burnetii antibodies, Leptospira antibodies, syphilis, aspergillus galactomannan antigen, and histoplasma antigen. Hypercoagulability work-up was also unremarkable and included DRVVT screening for lupus anticoagulant, anticardiolipin antibodies (×2), homocysteine levels, paroxysmal nocturnal hemoglobinuria screening, Protein C and S activity, antithrombin mutations, and factor V Leiden and prothrombin genotyping. Autoimmune work-up was also unyielding and included testing for antinuclear antibody, antineutrophil cytoplasmic antibody, antidouble-stranded DNA antibody, rheumatoid factor, cyclic citrullinated peptide, Sjogren antibody, myeloperoxidase antibodies, proteinase 3 autoantibodies, complement levels, Smith antibody, and RNP antibody. Given that there are no acute threat to limb or organ ischemia, the largely negative work-up, and no straight-forward mechanism to remove the aortic thrombus, we decided to manage the patient medically with close outpatient follow-up. She was started on heparin while inpatient which was transitioned to apixaban 5 mg twice a day and 81 mg of aspirin daily. This was done primarily because of the patient's preference to avoid frequent INR checks. A board-certified hematologist agreed with the use of apixaban with monthly outpatient follow-up to ensure patient compliance and clinical improvement. Patient was also counseled regarding smoking cessation. Catheter-based or surgical thromboembolectomy would have been considered if the patient would have developed acute limb ischemia or threatened organ.

### 3.3. One-Month Follow-Up

**Figure 6:**
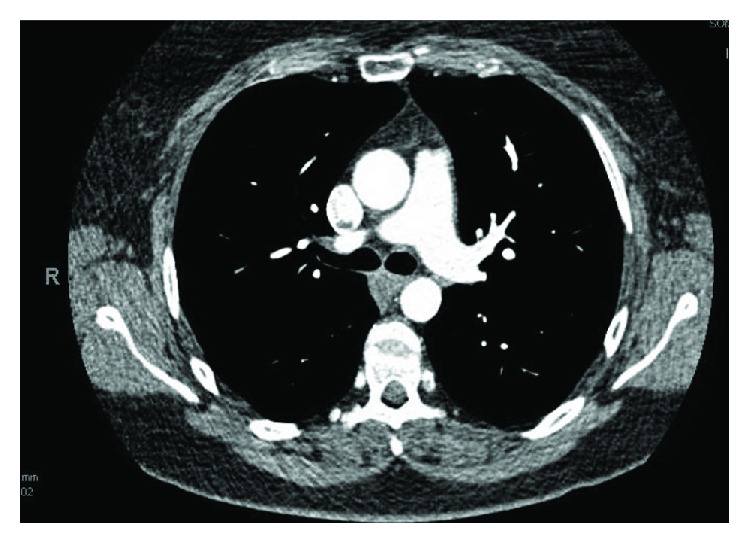
CT obtained for follow-up which reveals resolution of previously noted thrombus of the thoracic aorta.

At one month, the patient was seen in the clinic and denied symptoms of chest, back, or abdominal pain. She denied significant pain at rest or with exertion and did not exhibit any nonhealing wounds in either of her lower extremities. She noted being able to walk roughly one-quarter of a mile without symptoms prior to resting. She reported adherence with her daily aspirin and twice-daily Eliquis. At this time, arterial computed tomography of the abdomen and pelvis was repeated which revealed complete resolution of the filling defect involving the aortic isthmus and proximal descending aorta. There was no CT evidence to suggest vacuities. The spleen exhibited a lobulated superior margin with volume loss and the right kidney demonstrated a lobulated appearance of the right lower pole compatible with scarring from the previous infarct (Figure 6). The patient was instructed to continue with her antithrombotic regimen and follow-up in one month.

### 3.4. Two-Month Follow-Up

At this time, the patient no longer expressed having any symptoms of LE pain, swelling, or claudication. She reported being able to resume her daily activities with no limitations. Findings of the CTA were discussed with the patient at her two-month follow-up, and she was encouraged to continue with activity as tolerated. At this time, given the resolution of the mass after anticoagulation, it was felt that it was most likely an aortic thrombus. With regards the duration of therapy, we felt that this was likely unprovoked, and given it was arterial, we decided to continue anticoagulation for at least 12 more months pending further hematologic evaluation.

## 4. Discussion

Arterial thrombus is a rare clinical entity, particularly in the absence of aneurysm or severe atherosclerosis. The incidence of embolic events is higher in mobile pedunculated thrombi when compared to those that are layered and immobile (73% versus 12% risk of embolization, respectively) [7]. The most common manifestation includes consequences related to thromboembolic phenomena, as seen in our patient [2]. These complications are serious; therefore, early detection and management of their causes are imperative to patient care.

Aortic thrombi are typically secondary to aneurysmal disease, dissection, or severe atherosclerosis [5]. Alternative pathologies which may predispose one to being at risk for arterial thrombosis should always be investigated in efforts of preventing future thrombi and their sequela. Potential causes include trauma, malignancy, hypercoagulable states (factor V Leiden mutation, polycythemia, antithrombin III deficiency, protein C and/or S deficiency, etc.) and autoimmune disorders [2, 8–10]. Other risk factors including the presence of microscopic atherosclerotic disease (difficult to exclude clinically), smoking, and oral contraceptive use should also be addressed in patients as appropriate.

Distal embolization of the thrombus causes symptoms specific to the affected organ. Thromboembolic seeding to the renal vasculature can lead to acute kidney injury and failure depending on the renal mass distal to the blocked artery [11]. Complications of seeding to the spleen include, but are not limited to, left upper quadrant pain, anemia, leukocytosis, splenic pseudocyst, abscess, or hemorrhage [12]. Any of the four major types of acute mesenteric ischemic (acute superior mesenteric artery thromboembolic occlusion, mesenteric arterial thrombosis, mesenteric venous thrombosis, and nonocclusive mesenteric ischemia) are all potential complications of thrombus seeding to the colonic vasculature and can lead to significant morbidity and mortality [13]. Emboli seeding to the lower extremities can lead to acute limb ischemia which has an amputation rate of 13-15% and mortality rate of 9-12% [14].

Studies and guideline-directed management options are currently limited, and there have not been any prospective trials addressing therapeutic strategies. Options for treatment include anticoagulation, aortic surgery, thrombolytic therapy, and thromboaspiration [1, 15, 16]. The most common approach to initial management is anticoagulation [2, 5]. In patients with recurrence of distal arterial embolization or persistence of thrombus, aortic surgery may be considered [5, 17, 18]. Several studies have been published suggesting that aggressive management with aortic surgery may lead to less recurrence of distal embolization and complications including distal limb amputation particularly those in the ascending aorta or aortic arch [1, 2, 5, 19]. In our patient, given her vastly negative evaluation and lack of risk factors, we choose to purse management with apixaban and aspirin. Furthermore, there was significant concern for distal embolization given its location and mobility. To our knowledge, we are reporting one of the first case treated successfully with a non-vitamin K–dependent oral anticoagulant.

Our patient is unique in that a very extensive evaluation was unsuccessful in identifying the etiology of arterial thrombus formation. Our working diagnoses were broad throughout the case and included endocarditis, paradoxical embolism, cardiac neoplasm, and all potential causes of aortitis. After excluding infectious sources through an extensive evaluation and autoimmune conditions which may have led to aortitis, we decided to treat the patient empirically with apixaban and low-dose aspirin. Fortunately, our patient's thrombus resolved, and she experienced no residual symptoms at two months follow-up.

## 5. Conclusion

Primary aortic thrombus is a rare entity which has been previously described in the literature. It is a diagnosis of exclusion, and infectious, malignant, hypercoagulable, and autoimmune causes must be excluded. With regards to treatment, some studies recommend aggressive management to prevent risk of thrombus recurrence and arterial embolization while others recommend more conservative, less invasive medical management; however, there is no expert consensus or guidelines to direct therapy or its duration at this time. Given the lack of standardized guidelines at this time, we recommend a patient-specific approach which considers anticipated adherence to medications and follow-up, risk factors, results of laboratory work-up, and risk of further embolization. In low-risk patients, we propose that the use of a direct factor Xa inhibitors for oral anticoagulation may be an acceptable alternative to coumadin.

## Supplementary Materials

Supplementary Materials Images obtained by transesophageal echocardiogram of a large mass (thrombus) attached to the wall of the descending thoracic aorta.
